# Tracking fungal species-level responses in soil environments exposed to long-term warming and associated drying

**DOI:** 10.1093/femsle/fnad128

**Published:** 2023-12-06

**Authors:** Adriana L Romero-Olivares, Serita D Frey, Kathleen K Treseder

**Affiliations:** Department of Biology, New Mexico State University, Las Cruces, NM 88003, United States; Department of Natural Resources and the Environment, University of New Hampshire, Durham, NH 03824, Unites States; Department of Ecology and Evolutionary Biology, University of California, Irvine, CA 92697, United States

**Keywords:** fungi, catabolic processes, cell homeostasis, global climate change, metatranscriptomes, warming

## Abstract

Climate change is affecting fungal communities and their function in terrestrial ecosystems. Despite making progress in the understanding of how the fungal community responds to global change drivers in natural ecosystems, little is known on how fungi respond at the species level. Understanding how fungal species respond to global change drivers, such as warming, is critical, as it could reveal adaptation pathways to help us to better understand ecosystem functioning in response to global change. Here, we present a model study to track species-level responses of fungi to warming—and associated drying—in a decade-long global change field experiment; we focused on two free-living saprotrophic fungi which were found in high abundance in our site, *Mortierella* and *Penicillium*. Using microbiological isolation techniques, combined with whole genome sequencing of fungal isolates, and community level metatranscriptomics, we investigated transcription-level differences of functional categories and specific genes involved in catabolic processes, cell homeostasis, cell morphogenesis, DNA regulation and organization, and protein biosynthesis. We found that transcription-level responses were mostly species-specific but that under warming, both fungi consistently invested in the transcription of critical genes involved in catabolic processes, cell morphogenesis, and protein biosynthesis, likely allowing them to withstand a decade of chronic stress. Overall, our work supports the idea that fungi that invest in maintaining their catabolic rates and processes while growing and protecting their cells may survive under global climate change.

## Teaser

Inter-specific differences in the transcriptional response of fungi to global change/warming under field conditions.

## Introduction

Climate change is affecting soil microbial communities, and thus, the cycling of carbon (Melillo et al. 2002, Allison and Treseder 2011, Cavicchioli et al. 2019, IPCC 2021). Because soil microbes mediate biogeochemical cycles, their responses to global change drivers, such as warming and warming-induced drying (referred to as warmed and warming from here onwards), have resulted in ecosystem-scale impacts to the carbon cycle, including changes in decomposition and CO_2_ emissions (Allison and Treseder 2008, Melillo et al. 2017; Romero-Olivares et al. 2017a,b). These impacts are partially caused by community-level changes, where pathogenic and other weak-decomposer fungi (i.e. fungi with a limited suite of enzymes to break down organic matter) increase in abundance under warming, investing more resources in cell metabolic maintenance rather than in decay (e.g. Treseder et al. 2016, Solly et al. 2017, Morrison et al. 2019, Romero-Olivares et al. 2019). However, we know very little about how fungi at the species-level are responding and adapting to global change drivers. We know even less about what these responses and potential physiological and molecular adaptation pathways look like in natural soil microbial communities.

Fungal species-level responses to global change drivers have been documented in laboratory settings. For example, *Neurospora discreta* was experimentally evolved for 1500 generations under elevated temperature conditions, resulting in greater resource investment in respiration and spore production at the expense of biomass production (Romero-Olivares et al. 2015). Other shorter time scale studies found similar results; catabolic processes, such as growth and respiration, are impacted by elevated temperature (Malcolm et al. 2008, Crowther and Bradford 2013). Acclimation studies in *Neurospora crassa* revealed that when exposed to heat shock (i.e. temperature shift from 15°C to 42°C), *N. crassa* invested in the production of molecules for cell homeostasis, such as heat shock proteins, while arresting the production of cell morphogenesis proteins, such as actin and tubulin (Mohsenzadeh et al. 1998). Whether or how microbial species respond and/or adapt to warming in natural soil environments, remains largely unknown (DeAngelis et al. 2019).

Tracking species-level responses to warming in natural environments can offer insight into potential adaptation pathways. Here, we tracked species-level responses in a natural soil environment by mapping community level soil metatranscriptomes against the genome of two wild fungal species isolated from control conditions and warmed treatment soils in a long-term field warming experiment. We chose *Mortierella* spp. and *Penicillium swiecickii* (referred to as *Mortierella* and *Penicillium* hereinafter) for two main reasons. First, they were previously found to be the most abundant and presumably active species—based on transcript counts—in control conditions and warmed treatment soils alike (Romero-Olivares et al. 2019). Second, these fungi are free-living and easy to isolate and grow in culture compared to, for example, ectomycorrhizal fungi, which require a host. This meant that we were able to consistently isolate them from soil samples from both control conditions and warmed treatment soils. Since the fungal community shifts in composition in response to global change drivers (e.g. Treseder et al. 2016, Morrison et al. 2019), species that are highly abundant under control conditions may decrease in abundance or even disappear under treatment conditions, therefore, isolating the same fungal species from different soil samples is very challenging. Our objective was to investigate how individual fungal species respond to global change drivers in a natural soil environment to advance our understanding on fungal responses to climate change and to gain insight on potential adaptation pathways. Specifically, we investigated potential physiological changes at the species level in a natural soil environment exposed to global change drivers, which provides a more realistic overview of fungal responses to global climate change compared to studies done under controlled laboratory settings. We addressed our objective by asking the following questions, (i) What changes do *Mortierella* and *Penicillium* experience, at the transcription level, when exposed to warming in a natural soil environment? (ii) What functional pathways and genes are affected in each species in response to warming? (iii) Are there any impacts to gene regulation in response to warming? and (iv) How are *Mortierella* and *Penicillium* strategizing resource investment under warming*?*

## Materials and methods

Our field warming experiment was located in a mature black spruce (*Picea mariana*) forest in Delta Junction, Alaska, United States (63°55’N, 145°44’W). The onset of this experiment happened in the summer of 2005 (Allison and Treseder 2008). Briefly, greenhouses and neighboring control plots were established in pairs in a 1 km^2^ area; control plots were left untouched, while greenhouses (i.e. warmed treatment) warmed the soil passively during the growing season (May-September) using closed-top chambers (n = 4). The top plastic panel was removed (September-May) to allow snow fall to reach the plots. The air inside the greenhouses was 1.6°C higher, on average, compared to control plots. The soil temperature at a depth of 5 cm was 0.5°C higher inside the greenhouses compared to control plots. These increases in temperature are within the expected range for high latitude ecosystems under global climate change (IPCC 2021). During the growing season, gutters and tubing re-directed precipitation into the greenhouses to minimize drying. However, the warming treatment resulted in higher evapotranspiration and reduced soil moisture by 22%, on average (i.e. warming-induced drying). In the summer of 2015, we collected four soil cores from the top 10 cm from inside center of each greenhouse and control plots (332 cm^3^) (n = 4) and placed them inside a plastic sterile Whirl-Pak®. Approximately one gram of soil was immediately soaked in 5 ml LifeGuard^TM^ Soil Preservation Solution (Qiagen, catalog 12 868) for RNA extraction avoiding soil disturbance as much as possible, to prevent transcription level changes. The preserved soil solution and the soil samples were kept in a cooler with ice for 24 h and transferred to a −80°C freezer and 4°C refrigerator, respectively. The preserved soil solution and the soil samples were processed within a week of collection.

The protocol for extracting RNA and sequencing of metatranscriptomics was described in detail in Romero-Olivares and collaborators (2019). Briefly, the Joint Genome Institute (JGI) used rRNA depletion protocols to prepare paired-end libraries, which were then fragmented and reverse transcribed. The fragmented cDNA was treated with end-pair, A-tailing, adapter ligation, and 10 or 15 cycles of PCR and sequenced using a HiSeq 2500 system. Sequencing projects are deposited at the JGI with project ids: 1107–496, –499, –504, –507, –509, –514, –519, and –520.

Simultaneously, we carried out various isolation methods for culturing fungi from soil samples. Briefly, we prepared petri plates with malt extract agar (MEA) (20 g/L of agar, 5 g/L malt extract, 5 g/L yeast extract) and potato dextrose agar (PDA) (39 g/L of potato dextrose agar dehydrated, MP Biomedicals™) and proceed to isolate fungi by two different methods. The first method was sprinkling 0.5 g of soil directly onto the MEA and PDA plates. The second method was by dilution-to-extinction, where 1 g of soil was diluted in 10 mls of autoclaved water under sterile conditions and then diluted serially 5 times (1:10, 1:100, 1:1000, 1:10 000, 1:100 000). From each dilution, we used 50 µl to inoculate in MEA and PDA plates. This resulted in approximately 60 petri plates that we incubated under two different conditions: 30 petri plates were incubated at 22°C for 7 days, and 30 more petri plates were incubated at 10°C for 3 days to discourage growth of fast growers, and then moved to 22°C for 5 more days. We randomly selected 8 colonies from each plate (480 total colonies), inoculated them in PDA plates to obtain a clean individual colony, incubated at 22°C for 7 days, and extracted DNA using the CTAB method. We amplified the ITS region using ITS1-ITS4 primers (White et al. 1990) and sequenced the amplicons using Sanger sequencing. We obtained good quality sequence data for 341 isolates and used BLAST (Sayers et al. 2009) to determine identity. We identified 10 isolates of *Mortierella* and 17 of *Penicillium* from different control and warmed plots. Once we determined we had the same species (i.e. ≥99% similarity in the ITS region), we chose four isolates for our study (two from each species; one from warmed treatment and one from control conditions) and deposited sequences in NCBI GenBank (*Penicillium* control, accession number: MW474735; *Penicillium* warmed, accession number: MW474736; *Mortierella* control, accession number: MW474738; *Mortierella* warmed, accession number: MW474737). We sent high quality DNA of these four colonies to the JGI to sequence their whole genome. These sequencing projects are deposited at the JGI with project ids: 1144–747, -771, -787, -789.

Metatranscriptomes and whole genomes were quality trimmed by removing adapters with Trimmomatic (v 0.39) using ILLUMINA TruSeq3-PE adapters with sliding window 4:15 and dropping reads below 25 bases long (Bolger et al. 2014) and quality checked with FastQC (v 0.11.5) (Andrew 2010). We assembled genomes using SPAdes (v 3.13.1) (Bankevich et al. 2012), quality assessed with QUAST (v 4.5) (Gurevich et al. 2013), and indexed with STAR (v 2.7.5c) (Dobin et al. 2013). Metatranscriptomes were aligned and mapped to whole genomes using STAR (v 2.7.5c) with ‘twopassMode Basic’ due to a lack of annotated reference genomes (Dobin et al. 2013). We used Cufflinks (v 2.2.1) with the default normalization and false discovery rate to estimate transcript abundance and test for differential expression (Trapnell et al. 2010). This pipeline resulted in multiple tables including transcript counts for control and warmed treatment samples, fold change data (i.e. the degree of change of transcript counts between control and warming in relation to the mean of normalized counts), and DNA sequences for each transcript.

We manually blasted each transcript against the GenBank database to identify them (Sayers et al. 2009). We selected a consensus gene based on % identity (≥ 80%), alignment length (≥ 100 bp), and E-value (≤ 1e^−50^), with a few exceptions (i.e. E-values ≥ 1e^−50^) (Table S1). We categorized transcripts based on InterPro (Blum et al. 2020) as having functions related to catabolic processes, cell homeostasis, cell morphogenesis, DNA regulation and organization, or protein biosynthesis (Table 1). A subset of genes in *Mortierella* and *Penicillium* could not be identified because BLAST resulted in ‘hypothetical protein’ or ‘uncharacterized protein’. Thus, this subset of genes was left out of the analysis (listed as “unknown” in Table S1). We identified ATP synthase, cytochrome c oxidase, heat shock proteins, histones, NADH dehydrogenase, ribosomal proteins, and translation elongation factor as genes of interest since they were the genes that were transcribed the most (i.e. more than 20 different transcripts each).

**Table 1.** Description of functional categories used to categorize transcripts, as well as examples of proteins involved in those categories. The full list of genes included in our study, their transcripts, and encoded proteins, as well as their cell function and functional category, are listed in Table S1.

Functional Category	Genes encoding for transcripts that translate proteins for:	Example, proteins involved in:
Catabolic processes	The breakdown of complex molecules to transform them into simpler forms while releasing energy.	- Glycolysis - Krebs cycle - Breakdown of aminoacids
Cell homeostasis	The maintenance of balance within a cell.	- Chaperone activity - Transport of molecules - Regulatory proteins
Cell morphogenesis	The formation of cells, specifically those involved in structural maintenance and growth.	- Cell growth - Cell structure - Cell division
DNA regulation and organization	Processes involving the organization and regulation of nucleic acids in the cell.	- DNA replication - DNA packaging - Transcription factors
Protein biosynthesis	The production of proteins.	- Ribosomal proteins

We used lme4 and lmerTest package in R (Bates et al. 2015, Kuznetsova et al. 2017, R Core Team 2021) to carry out mixed models for each functional category, individually for specific genes of interest, and for gene expression. For each functional category, warmed treatment and species were fixed factor, plot was random factor, and transcript counts was the response variable; we used post hoc t-test to determine significant differences between species, warmed treatment, and functional category. For genes of interest, we ran individual models for each gene of interest in each species; warmed treatment was the fixed factor, plot was the random factor, and transcript count was the response variable. For gene expression data of functional categories, species was fixed factor, plot was random factor, and expression fold change was the response variable. For gene expression of genes of interest, we ran individual t-tests comparing up regulated fold change expression between species, as well as down regulated fold change expression between species. In all cases, we used *P* ≤ 0.05 as significant. Our analyses were non-parametric because we ranked all data. The scripts for bioinformatics and statistical tests were deposited at https://github.com/adriluromero/warming_metagene. Computations were performed on Premise, a central, shared HPC cluster at the University of New Hampshire.

## Results

**Figure 1.**
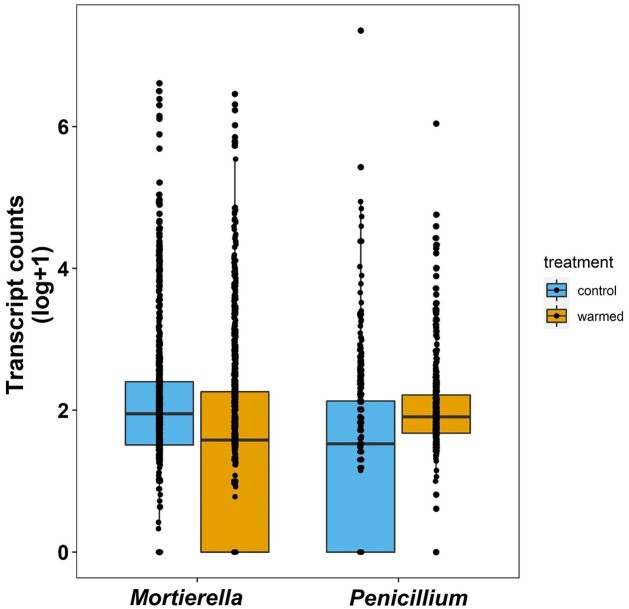
Total transcript counts in *Mortierella* and *Penicillium* in control and warmed samples. Box and whisker plots show the distribution of the data with lower and upper quartiles, mean, and lowest and highest observations plotted (n = 4). Each point represents the transcript count of a specific gene. Total transcript counts were not significantly different between control and warmed samples (*P* = 0.21).

**Figure 2.**
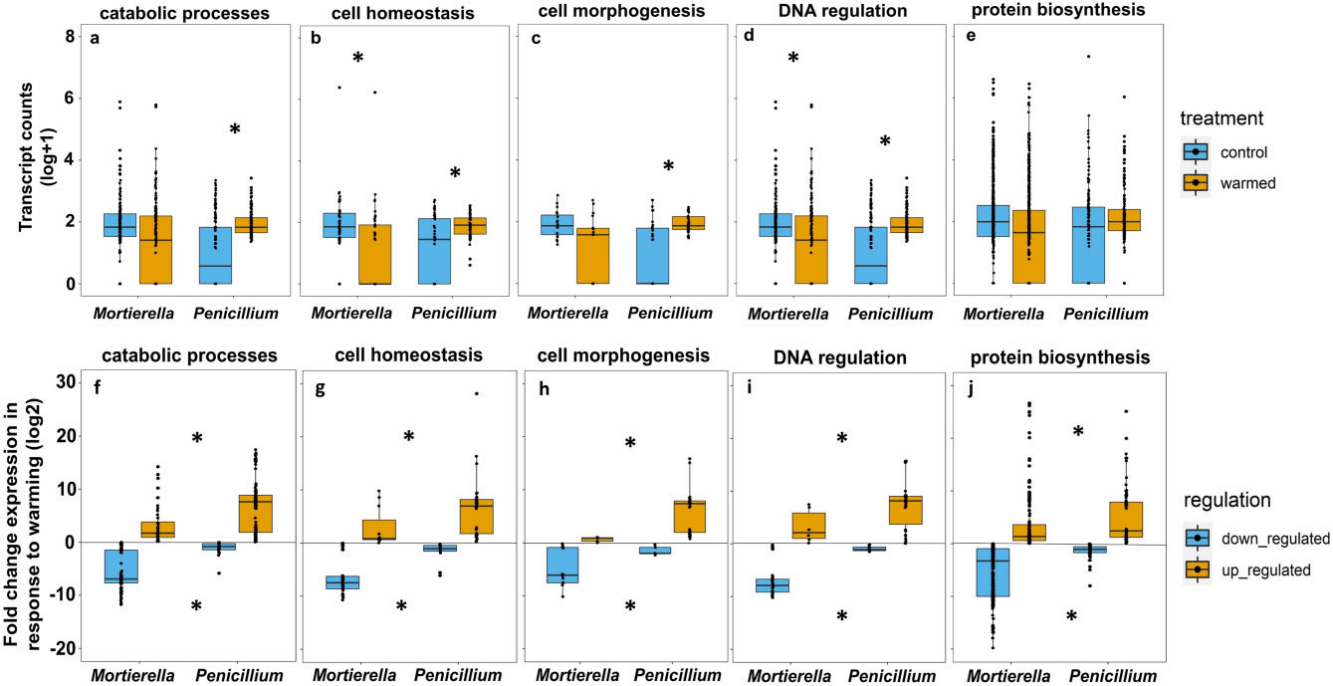
Transcript counts in *Mortierella* and *Penicillium* in control and warmed samples (a–e) and fold change expression in response to warming (f–j) by functional category. Box and whisker plots show the distribution of the data with lower and upper quartiles, mean, and lowest and highest observations plotted (n = 4). Each point represents the transcript count of a specific gene (a–e) and the fold change expression of a specific gene (f–j). Asterisks denote significance at *P* ≤ 0.05 between control and warmed samples within each species (a–e) and significance between fold change expression between species (f–j).

**Figure 3.**
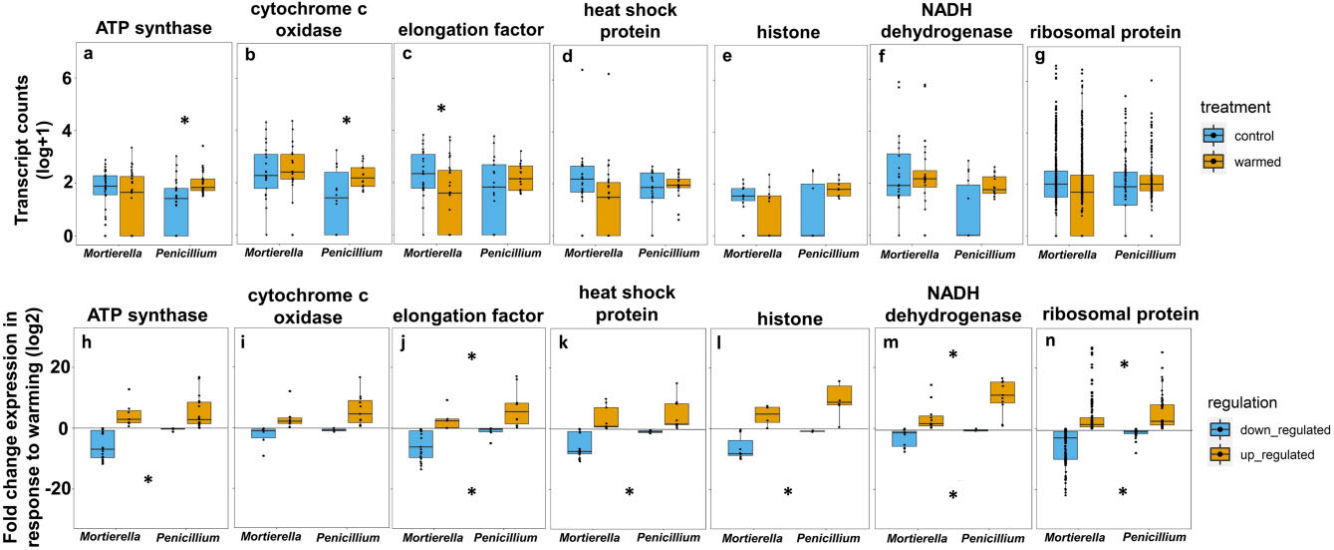
Transcript counts in *Mortierella* and *Penicillium* in control and warmed samples (a–g) and fold change expression in response to warming (h–n) in response to warming by genes of interest. Box and whisker plots show the distribution of the data with lower and upper quartiles, mean, and lowest and highest observations plotted (n = 4). Each point represents the transcript count of a specific gene (a–g) and the fold change expression of a specific gene (h–n). Asterisks denote significance at *P* ≤ 0.05 between control and warmed samples within each species (a–g) and significance between fold change expression between species (h–n).

To answer our first question, we found that there was no significant difference between overall transcript counts of *Mortierella* and *Penicillium* between control and warmed plots (*P* = 0.21), corroborating that these fungi were equally active under control and warmed conditions (Fig. 1). However, a breakdown of the transcript counts by functional category showed interspecific differences in the response of functional pathways and genes to warming. In all functional categories, except for protein biosynthesis, there was a significant interaction between species and warmed treatment, revealing that *Mortierella* and *Penicillium* responded differently to warming (catabolic processes, Fig. 2a, *P* = 0.05; cell homeostasis Fig. 2b, *P* < 0.01; cell morphogenesis Fig. 2c, *P* < 0.01; DNA regulation and organization Fig. 2d, *P* < 0.01; protein biosynthesis Fig. 2e, *P* = 0.60). Specifically, under warming, *Penicillium* had on average, significantly higher transcript counts for all functional categories, except protein biosynthesis, when compared to control conditions (catabolic processes, Fig. 2a, *P* < 0.01; cell homeostasis Fig. 2b, *P* = 0.04; cell morphogenesis Fig. 2c, *P* < 0.01; DNA regulation and organization Fig. 2d, *P* = 0.05; protein biosynthesis Fig. 2e, *P* = 0.64). In contrast, under warming, *Mortierella* had on average, significantly less transcript counts for cell homeostasis and DNA regulation and organization compared to control conditions (Fig. 2b, *P* < 0.01 and Fig. 2d, *P* = 0.01); but catabolic processes, cell morphogenesis, and protein biosynthesis were not significantly different between control and warmed samples (Fig. 2a, *P* = 0.39; Fig. 2c, *P* = 0.07; Fig. 2e, *P* = 0.24, respectively). Analyses for specific genes of interest showed that the transcription for ATP synthase and cytochrome c oxidase was significantly higher under warmed treatment compared to control conditions in *Penicillium* (Fig. 3a, *P* < 0.01 and Fig. 3b, *P =* 0.05, respectively), and that translation elongation factor was significantly lower in warmed treatment compared to control conditions in *Mortierella* (Fig. 3c, *P = 0.05*).

Moreover, our results show that although very few genes were significantly up or down regulated in response to warming (Table S1), *Mortierella*’s fold change expression was significantly down regulated at lower fold change compared to *Penicillium* in all functional categories (catabolic processes, Fig. 2f, *P* < 0.01; cell homeostasis Fig. 2g, *P* < 0.01; cell morphogenesis Fig. 2h, *P* < 0.01; DNA regulation and organization Fig. 2i, *P* < 0.01; protein biosynthesis Fig. 2j, *P* = 0.01). Whereas *Penicillium*’s fold change expression in response to warming was, on average, significantly upregulated at higher fold change compared to *Mortierella* (catabolic processes, Fig. 2f, *P* = 0.03; cell homeostasis Fig. 2g, *P* = 0.01; cell morphogenesis Fig. 2h, *P* < 0.01; DNA regulation and organization Fig. 2i, *P* = 0.02; protein biosynthesis Fig. 2j, *P* < 0.01). In other words, although both species had up and down regulated genes, *Mortierella* consistently downregulated at a lower fold change under control conditions compared to *Penicillium*, and *Penicillium* always upregulated at a higher fold change under control conditions compared to *Mortierella*. For specific genes of interest, fold change expression for all genes, except cytochrome c oxidase, was significantly down regulated at lower fold change in response to warming in *Mortierella* compared to *Penicillium* (ATP synthase, Fig. 3h, *P* < 0.01; cytochrome c oxidase, Fig. 3i, *P* = 0.25; elongation factor, 3j, *P* < 0.01; heat shock protein, 3k, *P* < 0.01; histone, 3l, *P* < 0.01; NADH dehydrogenase, 3 m, *P* < 0.01; ribosomal protein, 3n, *P* < 0.01). In addition, only NADH dehydrogenase and ribosomal proteins were significantly upregulated at higher fold change in *Penicillium* compared to *Mortierella* (Fig. 3m, *P* = 0.01 and Fig. 3n, *P* = 0.02, respectively).

**Figure 4.**
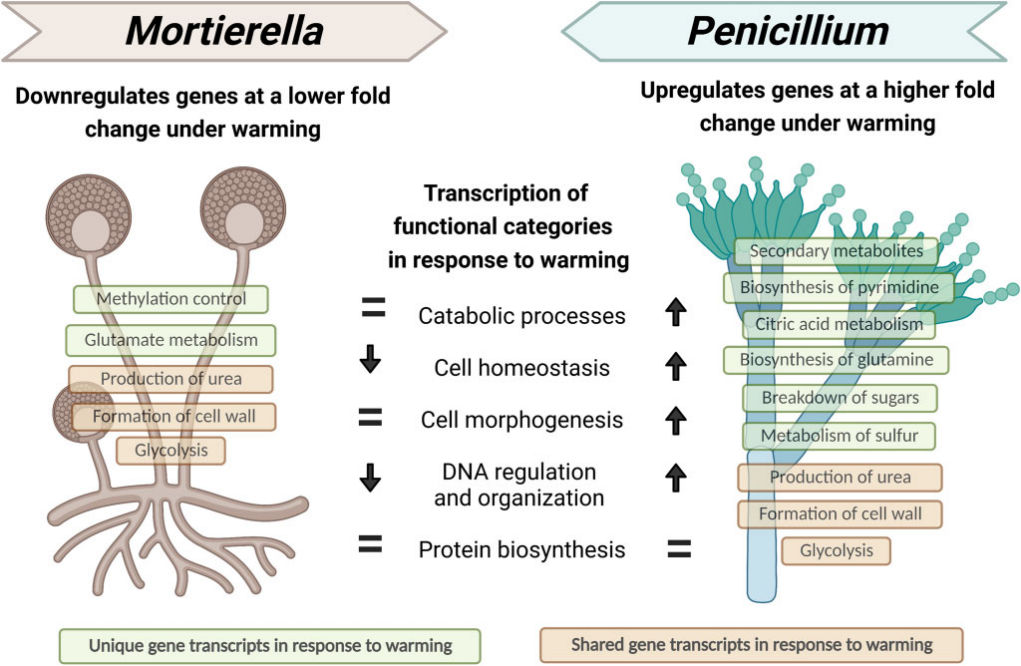
Summary of responses of *Mortierella* and *Penicillium* under warming. *Mortierella* downregulates at a lower fold change under warming, while *Penicillium* upregulates at a higher fold change under warming. Secondary metabolites, biosynthesis of pyrimidine, citric acid metabolism, biosynthesis of glutamine, breakdown of sugars, and metabolism of sulfur are a few examples of unique gene transcripts in *Penicillium*. Methylation control and glutamate metabolism are a few examples of unique gene transcripts in *Mortierella*. Contrastingly, production of urea, formation of cell wall, and glycolysis were gene transcripts present in both *Mortierella* and *Penicillium*. Illustration created with BioRender.com, license agreement UG24RGWD3U.

In terms of strategizing resource investment under warming, *Mortierella* and *Penicillium* again displayed significant differences (Fig. 4). In response to warming, *Mortierella* transcribed mostly genes involved in glutamate metabolism (1-pyrroline-5-carboxylate dehydrogenase) and methylation control (adenosylhomocysteinase). *Penicillium* transcribed many genes, including those involved in biosynthesis of pyrimidine (aspartate carbamoyltransferase), citric acid metabolism (citrate synthase), biosynthesis of glutamine (glutamine synthase), breakdown of sugars (glycoside hydrolase and transketolase), secondary metabolites (terpenoid synthase), breakdown of xylose (xylose reductase), and metabolism of sulfur (sulfite reductase) (Table S1). Some genes were transcribed by both fungal species but differed in their warming response. For example, both fungi transcribed genes involved in urea production and glycolysis, but *Penicillium* transcribed more under warming in contrast to *Mortierella*, which transcribed less. As such, fold change expression for these genes appeared as upregulated for *Penicillium* and down regulated for *Mortierella* (Table S1) (Fig. 4). Moreover, in response to warming, *Penicillium* transcribed genes involved in cell wall formation, such as 1,3-beta-glucanosyltransferase and α-glucan synthase, as well as membrane proteins, actin, and tubulin. *Mortierella* also transcribed genes for actin and tubulin but in lower abundance (Table S1).

## Discussion

Protein biosynthesis genes were the most abundant transcripts in *Penicillium* and *Mortierella* in both control conditions and warmed treatment (Fig. 2 and Table S1). Most protein biosynthesis transcripts in our data are involved in ribosome biogenesis (e.g. 40S and 60S ribosomal proteins). The production of ribosomes is an energy demanding process associated with rapid growth, but can also be associated with oxidative stress protection (Albert et al. 2019). Under control conditions, *Mortierella* and *Penicillium* may be transcribing ribosomal proteins for rapid growth, while under warmed conditions ribosomal proteins may be conferring protection to oxidative stress. Since *Mortierella* and *Penicillium* maintained and increased, respectively, their investment in catabolic processes under warming (Fig. 2a), reactive oxygen—a by-product of aerobic metabolism—may be accumulating in cells, causing oxidative stress (Shimizu 2018). Therefore, under warming, *Mortierella* and *Penicillium* may need to keep up with the production of ribosomes for protection against oxidative stress. This strategy may be especially needed for *Mortierella*, since antioxidant-related genes were transcribed at lower abundance under warming and drying (e.g. thioredoxin) (Table S1). Interestingly, ribosome biogenesis has been positively correlated with the ability of certain fungi to rapidly consume glucose through the fermentation pathway (Mullis et al. 2020). This relationship could allow them to keep growing through the consumption of sugars via low-efficiency fermentation (Mullis et al. 2020). Indeed, the ability to ferment, especially under aerobic conditions (i.e. Crabtree effect) has been associated with a selective advantage in yeast (Piškur et al. 2006). But the ability to ferment under aerobic conditions has also been documented in other fungi (e.g. Mullis et al. 2020). In our study, genes that may be related to the process of fermentation, such as zinc-dependent alcohol dehydrogenase (Raj et al. 2014), were transcribed at higher abundance under warming in *Penicillium* (Table S1). Although alcohol dehydrogenases have other functions that do not necessarily relate to fermentation, these results suggest that *Penicillium* could be fermenting to acquire energy under warming. If so, this strategy might be providing *Penicillium* with a competitive advantage over other fungi in response to the warming treatment.

Only two specific genes of interest, ATP synthase and cytochrome c oxidase, were transcribed more under warming compared to control conditions, and only in the case of *Penicillium* (Fig. 3). Even though the transcription of heat shock proteins, as a whole, was not significantly different between control conditions and warmed treatment (Fig. 3d), the transcription of heat shock protein 70 and 90, which are known to have a role in morphogenesis, heat stress, and pH stress (Tiwari et al. 2015) was highly upregulated in response to warming in both fungal species (Table S1). These results suggest that under warming, *Mortierella* and *Penicillium* may be investing in the production of protective molecules since they may have been experiencing heat stress and/or pH stress. Similar results have been reported in other fungi exposed to heat stress and/or pH stress. Specifically, *Schizophyllum commune* transcribed heat shock protein 70 and 90 after experiencing a shift in temperature from 21°C to 55°C (Higgins and Lilly 1993). Also, *Neurospora crassa* and *Aspergillus nidulans* transcribed genes for heat shock protein 70 and 90 in response to heat shock and extracellular pH changes (Mohsenzadeh et al. 1998, Squina et al. 2010, Freitas et al. 2011). Even though *Mortierella* and *Penicillium* experienced a relatively small temperature shift (∼0.5°C–1.5°C on average), these changes in abiotic conditions over a long period of time (∼10 years) may have been exerting chronic stress, resulting in the upregulation of certain heat shock protein genes in response to warming (Table S1). Although these proteins are known to be produced as a response to unfavorable conditions, biotic or abiotic, they also have a role in basic biological processes, such as gene transcription and protein translation (Tiwari et al. 2015). Aside from changes in gene expression, fungi may shift the function of heat shock proteins and use them for transcription and translation under control conditions, and for cell homeostasis and protection under warming. Accordingly, this shift in function would not change the number of transcripts between control and warmed samples. It has been proposed that the upregulation of specific genes plays a critical role in the retention of said genes (Zhang et al. 2019). Thus, further attention to upregulation of genes in wild fungal communities exposed to global change drivers may provide insight into adaptation strategies and traits that may be under selection.

We speculate that increases in transcription of most functional categories in *Penicillium* but not *Mortierella* (Fig. 2 and Table S1) could support the idea that even though both species were active under warming, *Penicillium* seemed to be thriving while *Mortierella* seemed to be surviving (Fig. 4). Specifically, *Penicillium* showed increased activity in most metabolic processes, except protein biosynthesis which remained unchanged (Fig. 2). In addition, we found evidence that that *Penicillium* may have been actively growing because it transcribed genes involved in cell wall and membrane formation. Contrastingly, *Mortierella* showed reduced activity in cell homeostasis and DNA regulation and organization, and no change in catabolic processes, cell morphogenesis, and protein biosynthesis which may indicate that *Mortierella* was investing in cell structure maintenance rather than growth. Altogether, this suggests that responses to warming, and thus, potential adaptation pathways, may be species-specific. However, both species either maintained or increased investment in catabolic processes, cell morphogenesis, and protein biosynthesis, suggesting that prioritizing those processes may be critical for their survival. But the fact that the total transcription activity of neither fungi differed between control conditions and warmed treatment suggests that the interspecific differences in functional gene transcription did not result in a change in total activity levels of the fungi (Fig. 1) and that both fungi have been able to survive a decade of chronic stress.

By studying two fungal species and their response to warming, we present a model study to track species-level responses to global change drivers in a natural soil environment. Our results represent a snapshot specific to the day and time when we collected soil. Thus, future studies should concentrate efforts on changes across time (i.e. minutes, days, months, years) as research has shown that microbial resource investment is highly dynamic and varies with season (Žifčáková et al. 2016, 2017). Considering micro-scale variations should also be a priority, as we found substantial plot variation (Fig. S1) which was probably the effect of plot-specific differences in soil conditions and/or plot microclimate. Even though these variations probably do not have an effect at the ecosystem scale in our work, the added effect of microscale interactions may give rise to large-scale effects on biogeochemical cycles (Kim and Or 2019, König et al. 2020). Moreover, future studies should explore more than two fungal species to provide a broader overview of the metabolic investment and potential adaptation strategies that fungi are undergoing when exposed to warming. Specifically, investigating how transcription changes in fungi that increase and decrease in abundance under warming may provide a good understanding of which genes provide a competitive advantage/disadvantage under stress. Similarly, investigating fungi with different ecological functions and focusing on functional genes, such as decomposition related genes (e.g. CAZy) (Lombard et al. 2014), provides an overview on how warming is affecting the fungal community more broadly, as well as the carbon cycling processes they mediate. Although we identified some CAZy transcripts in our dataset, these were not significantly different between the control conditions and warming treatment (Table S1). Finally, future studies will benefit from increased computational power in high-performance computer clusters and the development of memory-efficient software, as access to random access memory (i.e. RAM) limited the amount of samples that we could analyze (Romero-Olivares et al. 2019).

In conclusion, our work offers insight into how two fungal species are responding to warming in a natural soil environment. We present a model study, which can be replicated in other ecosystems, to track species-level responses in a natural soil environment and provide insight into the specific strategies that local fungal species undergo to ensure their survival under global climate change. We found evidence that investing in the transcription of critical genes involved in catabolic processes, cell morphogenesis, and protein biosynthesis under warming has allowed *Mortierella* and *Penicillium* to withstand over a decade of chronic stress. This suggests that investing in maintaining catabolic rates and processes while growing and protecting their cells may be a good strategy for fungi to survive under global climate change.

## Supplementary Material

fnad128_Supplemental_File
