# Supplementary material for: Tracking fungal species-level responses in soil environments exposed to long-term warming and associated drying
Source: FEMS Microbiol Lett. 2023 Dec 6;370:fnad128. doi: 10.1093/femsle/fnad128 (PMC10748604; doi:10.1093/femsle/fnad128)

Supplementary Figure 1.

Transcript counts in A) *Mortierella* and B) *Penicillium* in control and warmed samples by category and by plot. Box and whisker plots show the distribution of the data with lower and upper quartiles, mean, and lowest and highest observations plotted. Each point represents the transcript count of a specific gene.


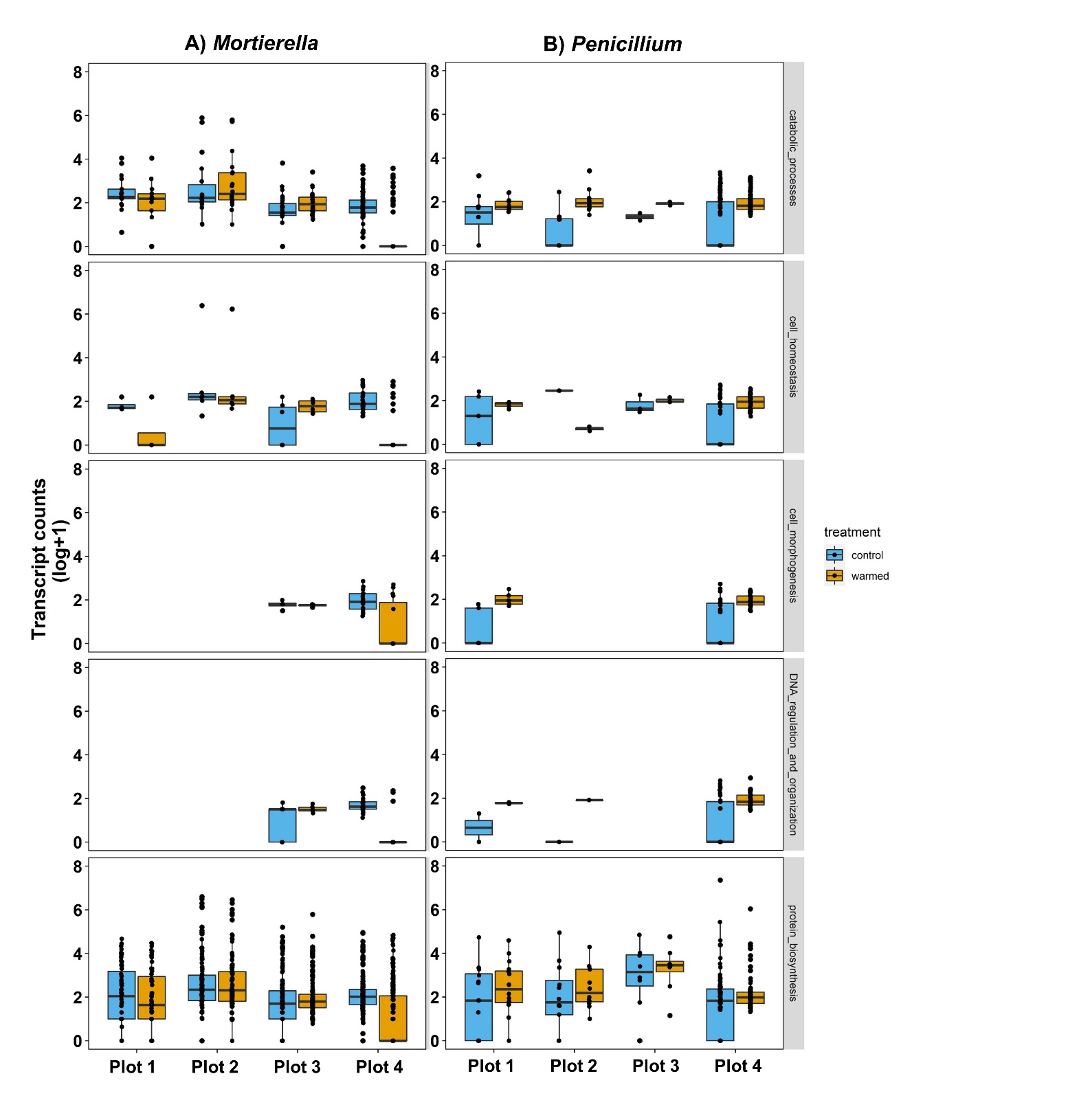

Supplement: fnad128_Supplemental_File [file fnad128_supplemental_file.zip › Supplementary Figure 1_FINAL.docx]
